# An Update on the Emerging Role of Visfatin in the Pathogenesis of Osteoarthritis and Pharmacological Intervention

**DOI:** 10.1155/2020/8303570

**Published:** 2020-08-08

**Authors:** Dong-Feng Han, Yang Li, Hui-Ying Xu, Rong-Hang Li, Ding Zhao

**Affiliations:** ^1^Department of Emergency Medicine, The First Hospital of Jilin University, Changchun, Jilin 130021, China; ^2^Department of Thoracic Surgery, The First Hospital of Jilin University, Changchun, Jilin 130021, China; ^3^Department of Ultrasound, The First Hospital of Jilin University, Changchun, Jilin 130021, China; ^4^Department of Orthopedic Surgery, The Second Hospital of Jilin University, Changchun, Jilin 130021, China; ^5^Department of Orthopedic Surgery, The First Hospital of Jilin University, Changchun, Jilin 130021, China

## Abstract

Osteoarthritis (OA) is one of the most common degenerative joint diseases that affects millions of people worldwide, mainly the aging population. Despite numerous published reports, little is known about the pathology of this disease, and no feasible treatment plan exists to stop OA progression. Recently, extensive basic and clinical studies have shown that adipokines play a key role in OA development. Moreover, some drugs associated with adipokines have shown chondroprotective and anti-inflammatory effects on OA. Visfatin has been shown to play a detrimental role in the progression of OA. It increases the production of matrix metalloproteinases and a disintegrin and metalloproteinase with thrombospondin motifs (ADAMTS), induces the production of interleukin (IL)-1*β*, IL-6, and tumor necrosis factor-*α*, affects the differentiation of mesenchymal stem cells to adipocytes, and induces osteophyte formation by inhibiting osteoclastogenesis. Although some side effects of chemical visfatin inhibitors have been reported, they were shown to be successful in the treatment of diabetes, cancer, and other diseases that can utilize Chinese herbs, further suggesting that similar therapeutic strategies could be used in OA prevention and treatment. Here, we describe the pathophysiological mechanism of visfatin in OA and discuss some potential pharmacological interventions using Chinese herbs.

## 1. Introduction

Osteoarthritis (OA) is one of the most common forms of arthritis and a major cause of disability in the elderly population [1, 2]. In the past, OA was generally attributed to mechanical processes, known as the “wear and tear” paradigm [3]. However, numerous cohort studies have reported that obesity is also a risk factor for OA in non-weight-bearing joints [4, 5]. In recent years, some studies have shown that adipokines, secreted by white adipose tissue (WAT), also play important roles in OA. For example, leptin and resistin induce the production of prostaglandin E (PGE), matrix metalloprotein (MMP) -1 and -13, and other inflammatory factors. On the contrary, adiponectin plays a protective role in OA by inhibiting the release of MMP-13 and upregulating the expression of type II and type X collagens [3, 6–9].

Adipokines have a systemic effect on the endocrine system and may be a vital link between obesity and OA [10]. Recently, there has been great interest in the role of the fat cell-derived protein, visfatin, in the pathophysiology of OA because it plays a crucial role in cartilage and bone homeostasis [11, 12]. The purpose of this review is to summarize the current knowledge on the pathophysiological mechanism of visfatin in OA and discuss potential pharmacological interventions.

## 2. Visfatin

Visfatin, discovered in 1994, is a 52 kD adipokine protein, also known as pre-B cell colony-enhancing factor (PBEF) and nicotinamide phosphoribosyltransferase (NAMPT) [13]. Visfatin promotes B cell maturation and is expressed widely in WAT and other tissues of humans [14, 15]. Haider et al. compared visfatin levels in lean and obese individuals and the effect of exercise training on its concentrations. They found that serum levels of visfatin increase in obese individuals and can be reduced by losing weight [16]. Although some researchers proved that visfatin shows the function of insulin-mimetic, the role of visfatin in glucose metabolism is still unclear [17–19]. Other studies show that visfatin induces chemotaxis and production of interleukins (IL) -1 and -6, as well as tumor necrosis factor-*α* (TNF-*α*) in lymphocytes of obese patients. This suggests that visfatin may be involved in obesity-associated inflammatory states [20].

## 3. Visfatin in OA Patients

In recent years, several researchers have measured visfatin levels in patients with OA. Some researchers revealed that circulating and local visfatin levels in these patients were higher than those in healthy controls (serum concentration: 36.3 vs. 27.3 ng/ml, *p* < 0.05; synovial fluid (SF): 8.95 vs. 4.48 ng/ml, *p* < 0.001) [12]. These results indicate that visfatin is involved in the pathophysiological process of OA, and articular tissues may affect the SF levels of visfatin [21].

Interestingly, cartilage and synovial tissues of patients with OA have been shown to secrete more visfatin than those of healthy subjects [22]. Moreover, the expression of visfatin in infrapatellar fat pad tissues of OA patients is higher than that in matched subcutaneous WAT [23]. Furthermore, visfatin has also been shown to be expressed in osteophytes by various articular cell types including osteoblasts, osteoclasts, and chondrocytes in patients with OA [24].

Levels of visfatin in serum or SF appear to be associated with lipid metabolism, inflammation, and progression of clinical disease [25]. Lee and Bae analyzed serum visfatin and C-reactive protein (an inflammatory marker) levels of 813 patients with rheumatoid arthritis and found a positive correlation. These results indicate that visfatin might be related to lipid metabolism and the inflammatory process [26].

Visfatin levels in SF are increased in patients with OA who show more radiographic evidence of joint damage compared to those with less disease severity. Specifically, Duan et al. reported that SF visfatin levels in K-L grade 4 are significantly elevated compared to those of K-L grade 3 (10.57 vs. 7.54 ng/ml, *p*=0.001) [12].

## 4. Roles of Visfatin in OA

Visfatin plays a detrimental role in extracellular matrix homeostasis [27]. Junker et al. revealed that collagen types II and X are significantly reduced in chondrocytes that are stimulated by visfatin for 24 h [24]. Furthermore, visfatin increases the production of matrix metalloproteinases (MMPs) and a disintegrin and metalloproteinase with thrombospondin motifs (ADAMTS). Gosset et al. reported that visfatin-treated mouse articular chondrocytes show increased expression of MMP-3, -13 and ADAMTS-4, -5 [22]. Yang et al. revealed that visfatin increases the mRNA levels and activities of MMP-3, -12, and -13 in human OA cartilage chondrocytes. They further reported that injecting visfatin into the knee joints of mice triggers cartilage destruction by increasing the production of cysteine proteases as well as MMP-3, -12, and -13 in cartilage tissue at both the gene and protein levels [28].

Visfatin exerts a proinflammatory effect during the progression of OA. As discussed earlier, visfatin induces the production of IL-1*β*, -6, and TNF-*α* in lymphocytes [20]. Amy et al. showed that visfatin enhances the biological effects of IL-1 by increasing the activity of MMPs, nitric oxide (NO) production, and proteoglycan release in cartilage and meniscus tissue [29].

Visfatin affects the differentiation of mesenchymal stem cells (MSCs) and the activity of osteoclasts. Li et al. found that MSC lineage fate determination is affected by cell energy metabolism and revealed a possible mechanism for senile osteoporosis, indicating that visfatin may affect MSC differentiation into adipocytes or osteoblasts [30]. Furthermore, Lali et al. found that IL-8 levels are significantly increased by visfatin during MSC differentiation, and these elevated levels induce the differentiation of human bone marrow cells into osteoclasts [31]. Apart from the effects on osteoblast proliferation and collagen synthesis, visfatin also plays a crucial role in osteoclast formation by inhibiting osteoclastogenesis, which indicates its role in osteophyte formation in the context of inflammatory diseases [20, 32]. However, contrary to the above findings, Venkateshaiah et al. showed that visfatin promotes osteoclast activity and myeloma cell growth in multiple myeloma owing to its enzymatic activity [33]. The reasons behind these effects are not clear, and the authors suggest that further studies are required to gain better insight.

## 5. Signaling Pathways of Visfatin

Although a visfatin receptor has not been identified, some researchers have shown that it can bind directly to the insulin receptor (IR) to exert biological effects in certain cell types such as human embryonic kidney 293 and A549 lung epithelial cells [34, 35]. The IR is expressed widely in humans and murine chondrocytes and plays a key role in the pathophysiological process of OA [22]. Furthermore, Huang et al. showed that visfatin binds to *β*1 integrin to induce the expression of stromal cell-derived factor-1 [36].

**Figure 1:**
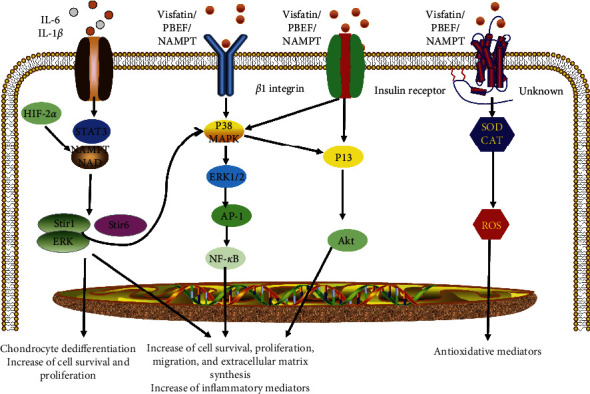
Signaling pathways of visfatin. First, visfatin signal is mediated by IL-6 and involves STAT-3, HIF-2*α*, NAMPT, SIRT1, and SIRT6 pathways. Second, visfatin signal is mediated by *β*1 integrin and involves ERK, p38 mitogen-activated protein kinase (MAPK), nuclear factor-*κ*B (NF-*κ*B), and AP-1 pathways. Third, visfatin signal is mediated by the IR and involves the PI3K, Akt, MAPK, and ERK1 pathways. Fourth, visfatin signal is demonstrated through the activation of an unknown receptor increasing the antioxidative enzymes superoxide dismutase (SOD) and catalase (CAT).

Despite the absence of an identified visfatin receptor, there are four signaling pathways associated with visfatin (Figure 1). The first visfatin signaling pathway is mediated by IL-6 and involves STAT-3, HIF-2*α*, NAMPT, SIRT1, and SIRT6 pathways. It has been reported that IL-6 trans-signaling affects the expression of visfatin, and the expression was mediated by STAT-3 and HIF-2 [37, 38]. Deacetylation of NAMPT by mammalian NAD+-dependent deacetylase, SIRT1, predisposed the protein to secretion by adipocytes. This is evident in a study with NAMPT mutants that reveals the deacetylation of lysine 53 and enhancement of NAMPT activity and secretion by SIRT1 [39]. Hong et al. also reported that visfatin could activate SIRT1. In response to IL-1*β*, SIRT1 activates extracellular signal-regulated kinase (ERK) and p38. The SIRT1-ERK complex participates in the dedifferentiation of chondrocytes induced by IL-1*β* through Sox9-mediated signals. Indeed, visfatin and ERK signaling could strengthen chondrocyte dedifferentiation mediated by SIRT1 [40].

SIRT1 has been shown to have a preventive role in OA by inducing cartilage-specific gene expression and inhibiting chondrocyte apoptosis [41, 42]. Interestingly, the data also show that visfatin increases NAD+ levels, thereby altering SIRT1 activity. Therefore, SIRT1, visfatin, and NAD+ have positive effects on human cartilage by increasing the expression of genes that encode the cartilage extracellular matrix [42].

The second visfatin signaling pathway is mediated by *β*1 integrin and involves ERK, p38 mitogen-activated protein kinase (MAPK), nuclear factor-*κ*B (NF-*κ*B), and AP-1 pathways. Liu et al. reported that visfatin could induce monocyte chemoattractant protein-1 and IL-6 production through p38, MAPK, and phosphoinositide 3-kinase (PI3K) pathways [43]. Moreover, Huang et al. showed that visfatin-induced stromal cell-derived factor-1 expression in colorectal cancer cells is mediated via the activation of the *β*1 integrin, ERK/p38, and NF-*κ*B/AP-1 pathways [36]. Lastly, Raghu et al. reported that visfatin induces the production of MMP-2 and -9 in human endothelial cells via the MAPK and PI3K/Akt signaling [44].

The third visfatin pathway is mediated by the IR and involves the PI3K, Akt, MAPK, and ERK1 pathways. Wang et al. revealed that visfatin stimulates proliferation and promotes the progression of the G1/S phase, as well as suppresses apoptosis in endometrial carcinoma tumor cells through the activation of IR, PI3K/Akt, and MAPK/ERK signaling pathways [45]. Brown et al. also showed that visfatin inhibits pancreatic *β*-cell apoptosis through MAPK/IR/PI3K signaling [46]. Moreover, Claire et al. showed that visfatin regulates the IR pathway activity to induce prostaglandin E2 release in chondrocytes [47].

The fourth signaling pathway involves redox reactions. Visfatin could increase the activity of superoxide dismutase to reduce the production of reactive oxygen metabolites [48]. Based on the protective regulation of this enzyme in cellular redox reactions and energy pathways, visfatin has been used as an effective target for cancer treatment [49].

## 6. Pharmacological Interventions

Visfatin plays an important role in cancer and inflammation; therefore, researchers have shown great interest in developing drugs and exploring traditional Chinese herbs.

Although various visfatin inhibitors have been developed, only three inhibitors (FK866, CHS828, and KPT-9274) have progressed to clinical trials. Although the trial data have not yet been published, some side effects have been reported in phase I/II clinical trials. These include thrombocytopenia, anemia, gastrointestinal toxicity, and hypoalbuminemia [50].

FK866, the most studied visfatin inhibitor, shows a high affinity for visfatin and prevents its biological activity by competing for the same binding site with visfatin [51]. In inflammatory arthritis in mice, the pharmacological inhibition of visfatin by FK866 decreased the severity of arthritis by reducing the expression of MMP-3, -13, and receptor or activator of NF-*κ*B ligand (RANKL) in vitro and in vivo [52]. Yang et al. established mouse models of OA by surgical destabilization of the medial menisci of the knee joints of rats or by using an intra-articular injection of visfatin. An intra-articular or intra-abdominal injection of FK866 (10 mg/kg) significantly inhibits cartilage destruction induced by visfatin and brings about a concomitant reduction of mRNA levels of the visfatin targets MMP-3, -12, and -13 in cartilage tissue [28].

The chemical derivatives of visfatin show a number of side effects; therefore, academic and industrial researchers have started to shift their interest toward traditional Chinese herbs. These outcomes have already been attained in diabetes, oncology, and other diseases using Chinese herbs, which suggest that similar strategies could be used to prevent and treat OA [53–56]. The mechanisms of action of Chinese herbs in regulating the biological effects of visfatin in arthritis require further study.

Curcumin is a natural compound extracted from the rhizome of turmeric that possesses antioxidant, anti-inflammatory, antiatherogenic, and antitumor activities [57]. Li et al. showed that curcumin can improve nonalcoholic fatty liver disease (NAFLD) by reducing the expression of visfatin in NAFLD rats [58]. In some cancer, the same results were found about curcumin which could decrease the expression of visfatin [59]. Furthermore, it suppresses synovial inflammation, oxidative stress, and matrix degradation of inflammatory chondrocytes through the Nrf2/ARE and NF-*κ*B signaling pathways [60]. In clinical trials, curcumin has proven to be effective in treating patients with OA and improving their Western Ontario and McMaster Universities Osteoarthritis Index (WOMAC) scores [61].

Emodin, an active component of *Rheum palmatum* and other Chinese herbs including *Polygonum cuspidatum*, *Polygonum multiflorum*, and *Cassia obtusifolia*, has been shown to possess anti-inflammatory, antiatherogenic, and antitumor activities [55]. Cui et al. showed that emodin alleviates severe acute pancreatitis (SAP) that is associated with acute lung injury (ALI) by decreasing visfatin expression and promoting polymorphonuclear neutrophil apoptosis [62]. In OA, emodin could protect cartilage from degradation by suppressing NF-*κ*B and Wnt/*β*-catenin signaling, which has been observed in cellular and animal experiments [63].

Genistein, a primary active ingredient of *Sophora japonica* and Sandougen, is known to inhibit inflammatory responses, reduce oxidative stress-induced damage, and exert anticancer effects [64]. Zhu et al. studied whether genistein protected alveolar epithelial cells from LPS-induced injury and reported that genistein plays a protective effect in lung injury by suppressing the activation of NF-*κ*B and alleviating the inflammatory response [65]. In OA, genistein suppresses the expression of NF-*κ*B activated by inflammatory cytokines and plays a protective role in preventing condylar cartilage damage in OA in rats [66].

Polydatin, a well-known component obtained from Rhizoma Polygoni Cuspidati, is known to have various pharmacological effects and shows anti-inflammatory, blood lipid-regulating, cholesterol lowering, and antishock properties [67]. Studies by Huang et al. revealed that polydatin can relieve atherosclerosis injury in mice through the downregulation of visfatin and inhibition of visfatin-inducing cholesterol deposits in macrophages [54]. In OA, polydatin protects the cartilage from degeneration by activating chondrocyte autophagic flux via the MAPK and PI3K/Akt signaling pathways. Furthermore, polydatin could inhibit inflammation reactions induced by IL-1*β* in chondrocytes through the activation of the Nrf2 signaling pathway [68, 69].

Additionally, other primary active ingredients of traditional Chinese herbs such as Russelioside B (RB) and salidroside have previously been reported to show antidiabetic, anticancer, anti-inflammatory, antishock, and antihyperlipidemic activities. Thus, these compounds may play an important role in protecting joints by regulating visfatin levels [53, 70].

## 7. Conclusion

At present, most evidence shows that visfatin plays a proinflammatory role in OA. The increasing interest in visfatin has gradually led to the uncovering of the intricate adipokine-mediated relationship between WAT and OA. Although many aspects are still unclear, this review highlights the molecular functions and mechanisms of visfatin in OA and discusses some of the potential pharmacological interventions using Chinese herbs. However, further investigations are necessary to fully understand the role of visfatin in OA.
